# Training Informal Supporters to Improve Responses to Victim-Survivors of Domestic Violence and Abuse: A Systematic Review

**DOI:** 10.1177/15248380231189191

**Published:** 2023-08-31

**Authors:** Karen Schucan Bird, Nicola Stokes, Carol Rivas, Martha Tomlinson, Mollin Delve, Lindsay Gordon, Alison Gregory, Kate Lawrence, Nicola O’Reilly

**Affiliations:** 1 University College London, London, UK; 2 SafeLives, Bristol, UK; 3 PHOEBE, Ipswich, UK; 4 University of Bristol, Bristol, UK; 5 Home-Start East Sussex, Newhaven, UK

**Keywords:** domestic violence, intervention/treatment, disclosure of domestic violence, cultural contexts

## Abstract

Informal supporters (friends, family, colleagues, and community members) play a crucial role in societal-wide responses to victim-survivors of domestic violence and abuse. Familial and social networks, however, report a sense of helplessness and difficulties in knowing how to respond. This mixed method systematic review examines the effectiveness, and perceived effectiveness, of training informal supporters to improve their responses to victim-survivors. A novel conceptual framework was developed to underpin the review. A systematic search of four electronic databases, specialist repositories, and websites were used to identify empirical research (in academic or gray literature). Eleven included studies examined educational interventions that aimed to improve responses from informal supporters. Quality appraisal was undertaken, and studies were judged to be “good enough” for synthesis. The studies in the review indicated that informal supporters recognized the value of training for building understanding and equipping them with the skills to respond to victim-survivors. The synthesis identified statistically significant improvements in the knowledge and attitudes of informal supporters in the immediate and short-term following training. Using a behavior change model to frame the evidence, the review found that training/educational activities prime informal supporters to respond to victim-survivors, as well as enhancing their capacity and motivation to do so. This increases the likelihood that informal supporters will take action to support victim-survivors of abuse. We don’t know, however, what type of support they will provide and/or whether it would be judged to be helpful by victim-survivors.

Friends, family, colleagues, and community members play an important role in supporting victim-survivors during and after experiences of domestic violence and abuse (DVA). Such individuals and groups serve as “informal networks” (Klein, 2012) that provide various forms of “informal social support” including practical assistance, emotional support, and/or resources (Budde & Schene, 2004). Such support can be crucial for meeting the immediate and longer term needs of victim-survivors, especially for those who face difficulties accessing formal services (e.g., insecure immigration status), have poor experiences of statutory agencies (e.g., racial discrimination), or fear negative consequences of seeking help from formal agencies (e.g., retribution from perpetrator) (Goodman, Epstein, et al., 2022; Sultana et al., 2022). In comparison to formal services, informal networks are uniquely placed to offer ongoing, mutual relationships of trust and care (Goodman, Epstein, et al., 2022; McKenzie et al., 2020) with scope to provide different types of support that can be tailored to the victim-survivor and their personal situation (Bellotti et al., 2021; Goodman, Banyard, et al., 2016). The majority of victim-survivors disclose their experiences of abuse within their relationship to at least one informal supporter (Johnson & Belenko, 2021; Sylaska & Edwards, 2014) and 35% of victim-survivors credit such support with helping them to leave, when desired (European Union Agency for Fundamental Rights, 2015). Wider evidence suggests that positive responses from familial and social networks can lead to improved outcomes for victim-survivors in terms of their help-seeking, mental well-being and physical health (Beeble et al., 2009; Coker et al., 2002; Nolet et al., 2021; Sylaska & Edwards, 2014; Zapor et al., 2018). The importance of informal social networks has been magnified by the pandemic (Gregory & Williamson, 2021; Sánchez et al., 2020) as formal service providers have struggled to make and maintain contact with victim-survivors (Esposito & Szypulska, 2022) and COVID mitigation policies have increased women’s vulnerability to abuse (Nordhues et al., 2021).

However, informal supporters may be reluctant or unable to support victim-survivors. Factors that inhibit the provision of support are multiple (Latta & Goodman, 2011) including informal supporters’ fear of retaliation from the perpetrator and concern for their own safety (Melgar et al., 2021), a sense of helplessness (Goodkind et al., 2003), and/or difficulties in knowing how to respond (Gregory, Feder et al., 2017; Latta & Goodman, 2011; McKenzie et al., 2020). Moreover, studies report that friends, family, colleagues, and community members may not always respond in a helpful manner. Expressions of doubt, blaming the victim and/or withdrawing support are identified as negative responses by victim-survivors (Nolet et al., 2021). Such reactions are subsequently associated with poorer mental health of the victim-survivor, a reduction in their wellbeing (Dworkin et al., 2019; Femi-Ajao et al., 2020; Sylaska & Edwards, 2014) and lower willingness to maintain social networks or further confide in friends or family (Nolet et al., 2021; Rose & Campbell, 2000).

Therefore, it is imperative to develop and implement interventions that enable informal supporters to respond positively to disclosures of abuse (Edwards & Dardis, 2020; Goodman, Banyard, et al., 2016; Ullman, 2021). Victim-survivors have defined such responses in terms of empathetic listening, emotional support, and/or practical help (Nolet et al., 2021; Sylaska & Edwards, 2014). Education and training can play a critical role in fostering empathy and teaching friends, family, colleagues, and community members how to respond (Edwards & Dardis, 2020; Ullman, 2021). In the UK, there are currently various educational and/or information resources tailored toward informal supporters ranging from online guidance about what to say to training programs and toolkits for employers. Yet, there is limited understanding of the outcomes or perceived effects of such interventions.

To date, there has been no attempt to systematically describe or synthesize the primary evidence base pertaining to educational activities aimed at friends, family, colleagues, and community members of victim-survivors of DVA. Existing systematic reviews have primarily focused on training in formal contexts, reporting promising improvements in understanding and recognition of DVA among healthcare professionals (Alshammari et al., 2018; Turner et al., 2017; Zaher et al., 2014). Only one systematic review has focused on training for informal supporters, but this considered a broad range of interventions that often included formal mechanisms of support alongside educational activities (Ogbe et al., 2020). Wider systematic reviews on informal support have focused on disclosure and help-seeking (such as Bundock et al., 2020; Femi-Ajao et al., 2020), social reactions (Dworkin et al., 2019; Ullman, 2021), or outcomes for supporters (Gregory, Williamson et al., 2017), but few focus on interventions. There is also an absence of underpinning conceptual models that explain the role played by informal social support interventions, alongside wider DVA service provision, in contributing to improvements in outcomes for victim-survivors (Goodman, Banyard, et al., 2016; Sullivan, 2018).

This article fills these gaps by advancing knowledge in two main ways: (a) developing a theoretical framework that explains how we might expect education/training for informal supporters to lead to improved outcomes for victim-survivors, (b) evaluating the effects and perceived effects of education/training for informal supporters in terms of cognitive and behavioral outcomes.

## Method

### Aim and Design

A mixed method review (Grant & Booth, 2009) aimed to examine effectiveness, and perceived effectiveness, of education/training for informal supporters. Recognizing the dearth of rigorous intervention studies in DVA (Bell & Coates, 2022; Feder et al., 2011) and the prominence of research from non-governmental organizations (Konya et al., 2020), the review aimed to include diverse study designs and maximize available data (Schucan Bird et al., 2023). This meant that the review recognized the value and contribution of quantitative and qualitative data, including from the gray literature, following precedent set by previous reviews of informal support interventions (Konya et al., 2020; Ogbe et al., 2020).

### Theoretical Framework

In the absence of a pre-existing theory of how educational interventions might lead to improved outcomes for victim-survivors, a conceptual framework was developed. A set of outcomes, and their relationships, were derived from existing reviews of educational training in DVA (Alshammari et al., 2018; Turner et al., 2017), empirical primary studies on wider informal social support interventions (identified in a sister project, see Schucan Bird et al., 2022), and systematic reviews reporting the impacts associated with informal social support (Nolet et al., 2021; Sylaska & Edwards, 2014). The initial framework was modified in response to feedback from the Advisory Group (see Stakeholder Engagement) and linked to a behavioral change model: Capability, Opportunity, Motivation, Behavior (COM-B) (Michie et al., 2011). According to this model, behavior change is associated with interventions that target and enhance three essential conditions: capability (“the individual’s psychological and physical capacity to engage in the activity concerned”), opportunity (“all the factors that lie outside the individual that make the behaviour possible or prompt it”), and motivation (“all those brain processes that energize and direct behaviour”). This model was linked with the theoretical framework to elucidate the role of different cognitive outcomes in effecting behavior change.

**Figure 1.**
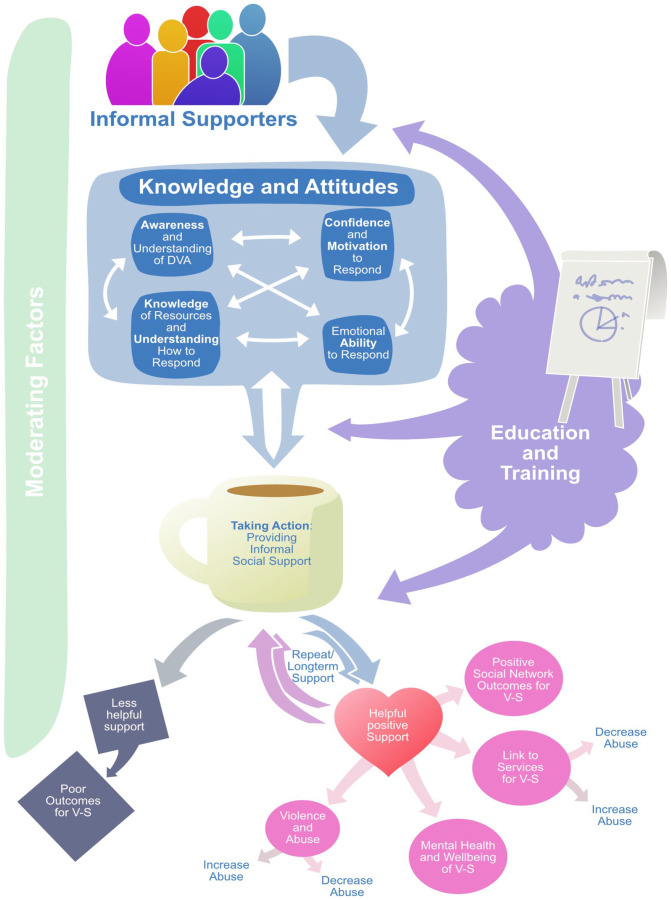
Logic model: How education/training interventions influence outcomes.

The theoretical framework (see Figure 1) recognizes that education/training for informal supporters is expected to improve four distinct, but interacting, cognitive outcomes (“knowledge and attitudes”) which align with the conditions specified by the COM-B behavior change model. Increases in knowledge of resources, for example, improves the *capability* of informal supporters to respond to disclosures of abuse while greater understanding of DVA increases their *opportunity* to recognize abuse in the first place. Training that heightens informal supporters’ awareness of the prevalence and impacts of DVA enhances their *motivation* to respond. Educational interventions need to simultaneously foster capability, opportunity, and motivation (COM) to empower informal supporters to take action (behavior change). Following training, such actions are expected to provide helpful, positive forms of support for victim-survivors (e.g., non-judgmental listening and emotional support) (Nolet et al., 2021; Sylaska & Edwards, 2014), that serve as a protective factor against exposure to abuse (Goodman, Dutton, et al., 2005) and improve their mental health, help-seeking, and longer term recovery (Sylaska & Edwards, 2014; Zapor et al., 2018). However, there is also scope for negative outcomes. Abuse can continue and potentially escalate following intervention by an informal supporter (McKenzie et al., 2020) or wider service providers such as the police or criminal justice system (Saxton et al., 2021). Further, the provision of negative, unhelpful responses despite educational intervention (e.g., forgetting training, DVA myths continue) may also lead to poor outcomes for the victim-survivor.

As illustrated in Figure 1, education/training can take place before, during, or after an informal supporter has responded to a victim-survivor with several potential feedback loops influencing outcomes (Liang et al., 2005). “Taking action” subsequently shapes informal supporters’ knowledge of, and attitudes toward DVA (e.g., improved understanding of the nature of abuse while providing support, e.g., Gregory, Williamson et al., 2017; Latta & Goodman, 2011; McKenzie et al., 2020). Similarly, positive forms of support for victim-survivors are associated with closer relationships and so engender longer-term, repeat provisions of support from the informal supporter (Edwards & Dardis, 2020; McKenzie et al., 2020). The effectiveness of education/training is expected to be moderated by many wider factors, such as geographical contexts (e.g., urban vs. rural Lanier & Maume, 2009) or gendered processes that shape abuse and informal responses (Klein, 2012; Liang et al., 2005).

### Stakeholder Engagement

An Advisory Group was created at the outset, composed of a diverse group of stakeholders including two individuals with lived experience, two frontline DVA service providers, and two DVA specialists in order to represent different types of knowledge/experience in the review process (Rees & Oliver, 2017). Three online meetings were held over the course of the review to refine the theoretical framework and define outcomes, identify priorities for in-depth analysis of studies, and shape review conclusions/messaging.

### Search Strategy

A broad search was devised to identify all studies on informal social support interventions, from which interventions focusing on education/training for social networks were identified. The strategy included multiple search sources. Four electronic databases were searched: ASSIA, PsychInfo, PubMed, and Social Policy and Practice. Search strings were developed, informed by similar reviews (Gregory, Williamson et al., 2017; Ogbe et al., 2020), that combined the concept of DVA (including *domestic violence*, *domestic abuse*, *intimate partner violence*) with informal social support (including *social support*, *social network*, *support system*). Specialist international databases of systematic reviews (Social Systems Evidence and Campbell Collaboration), policy-orientated research (World Health Organization and European Commission), and DVA reports (National Resource Centre on Domestic Violence, World Health Organization Violence Against Women Database) were also searched. The websites of domestic abuse organizations in the UK were handsearched by one reviewer. The list of organizations was compiled by members of the research team who were from the DVA sector and confirmed with the wider Advisory Group to ensure the coverage and credibility of the handsearch.

### Inclusion and Exclusion Criteria

The review included any empirical primary research that examined education/training interventions explicitly tailored toward informal supporters. Interventions were deemed eligible when the curriculum aimed to improve the response from friends, colleagues, or community members, current non-abusive partners, or any family member (including step-family, non-blood relatives, family-in-law) of the victim-survivor of DVA (developed from Gregory, Feder et al., 2017 definition). No date limits were set. Studies were excluded if the sample did not include victim-survivors or informal supporters, or separate data was not reported for these groups. Only publications reported in English were eligible for inclusion in the review. Screening was initially undertaken on title and abstracts, followed by full text. For each of these screening phases, a sample of references were screened independently by two researchers until a high level of consistency was reached. Decisions on complex studies were discussed and resolved by the whole team.

### Quality Assessment Method

All included studies were appraised using the Mixed Method Appraisal Tool (MMAT) (Hong et al., 2018). In addition, gray literature reports were judged according to the ACCODS checklist (Tyndall, 2010). Both tools were applied independently by two reviewers who then reached agreement on the overall judgment. A principle of “good enough” quality (Stewart et al., 2010) was used to decide whether and how studies could contribute to the synthesis. To do so, studies were tabulated to identify the overall MMAT judgment (% of relevant criteria fulfilled) together with the associated strengths and weaknesses of the designs based on this tool. “Good enough” studies included those that met 40% of the MMAT criteria and/or were deemed to offer significant and authoritative contributions to the field of informal social support based on the ACCODS tool. The potential contribution of gray literature reflects the wider value attributed to credible sources (in terms of respected colleagues or organizations working in DVA) in the sector (Casey et al., 2020).

### Data Extraction and Synthesis

A set of data extraction codes were applied to each study to capture details about the methods, sample, intervention, and findings. Two reviewers undertook the data extraction independently and agreed upon a final version. Discussions with a third reviewer were undertaken to resolve coding disagreements. The theoretical framework was used to guide the extraction for both quantitative and qualitative results. A dearth of studies together with variation in interventions (nature and scope) and poor reporting meant that quantitative meta synthesis was inappropriate. All studies judged to be “good enough” were reported narratively and grouped according to outcome and type of informal supporter. A preliminary synthesis was undertaken by the lead reviewer and further developed in collaboration with the wider team.

The COM-B framework (Michie et al., 2011) was then used to frame the evidence and draw conclusions about the effectiveness of education/training for shaping informal supporters’ response. In the absence of data from experimental study designs (Feder et al., 2011), incorporating principles of behavior change into educational interventions in DVA is helpful to assess the likelihood of future actions (Sammut et al., 2021). To do so, outcomes were categorized into COM with the associated evidence from included studies assessed through tabulation.

## Findings

### Characteristics of Included Studies

**Figure 2.**
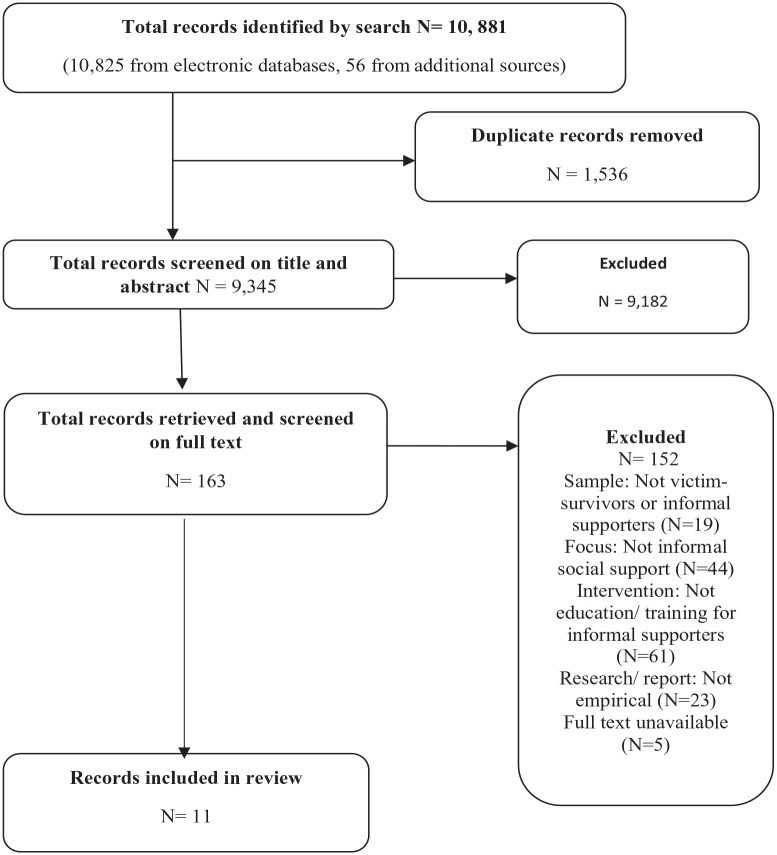
Flow of records through the review.

Of the 9,345 records initially found through our search strategy, the screening process identified 11 studies eligible for inclusion in the review (see Figure 2).

Included studies were conducted all over the world, five in North America, three in Europe, one in Vietnam and one in Australia. One study was described as “global” in reach (see Table 1). Study samples varied by the type of informal supporter and demographic make-up. Only one study included a sample with a balanced ratio of male and female supporters, drawn from supervisors at workplaces in Oregon, USA (Glass et al., 2010). Two studies included predominantly male supporters from one ethnic group, representing the targeted population groups and the compulsory nature of the training for Faith Leaders from Korean/American Korean churches (Choi et al., 2019) and Seventh-Day Adventist pastors (Drumm et al., 2018). Women-only samples were included in four studies: one targeting female peer supporters (Ross, 2013), two focusing on mothers (Prosman et al., 2014; Taft et al., 2011), and one included employees who had chosen to attend training (Debbonaire et al., 2011). Four studies included informal supporters from unspecified “diverse backgrounds” (Taft et al., 2011) or ethnic minority groups (Choi et al., 2019; Ross, 2013; Women’s Aid, 2020). Three of the 11 studies reported that some of the trained informal supporters had prior personal experience of DVA (Ross, 2013; Taft et al., 2011; Women’s Aid, 2020).

**Table 1.** Characteristics of Included Studies.

Author (Year) Study design, Country	Study Population/Sample	Education/Training Intervention	Summary of Findings (Outcomes/Themes Reported, Timing and Instruments)
Choi et al. (2019) Randomized Controlled Trial, USA	Informal supporters (Faith Leaders) Total sample *n* = 54 Gender: Female 24%, Male 76%; Ethnic/racial minority (Korean or Korean American) 100%	Compulsory attendance **Delivery**: Online **Format**: Presentation/lecture **Duration**: Between 1.5 and 3 hrs **Content**: Understanding DVA within Korean community and how to respond **Trainer**: Unspecified	**Cognitive** *Awareness & understanding of DVA* **3 months:** improvements in attitudes (η_p_^2^ = 0.08, p = .044) and knowledge (η_p_^2^ = 0.08, p = .074) of trained versus untrained *Knowledge of support services* **3 months:** knowledge of resources (η_p_^2^ = .11, *p* = .013) of trained versus untrained *Confidence and motivation* **3 months:** improvements in confidence in their ability to respond (η_p_^2^ = 0.01, *p* = .439) **Behavioral** *Provision of informal support* **3 months:** increase in actions taken by clergy who had received training, compared to those who had not, but the differences were not statistically significant (η_p_^2^ = 0.01, *p* = .409) **Instruments:** Unvalidated, researcher developed
Debbonaire et al. (2011) Before and After, Qualitative interviews and questionnaires UK	Informal supporters (Workplace colleagues) Total sample *n* = 28 (T1 questionnaires), *n* = 8 (T2 questionnaires and interviews) Female (100%) Ethnicity not reported	Voluntary attendance **Delivery**: Face to Face **Format**: Presentation/lecture **Duration**: 2-day train the trainers session for Human Resources professionals, “short” briefings for managers **Content**: Not reported **Trainer**: Domestic abuse organization ran training for HR; HR ran training for managers Multi-component intervention	**Cognitive** *Awareness & understanding of DVA* **6 months** Recognizing DVA: Bringing “the issue of domestic violence affecting staff into the open” (p. 8) *Knowledge of support services* **6 months** Practical responses: knowing “how to help staff experiencing domestic violence” (p. 10) Making connections: “other policies and work tools” *Confidence and motivation* **6 months** Confidence: “The domestic violence training gave me confidence” (p. 13), “more confident to provide support again” (p. 17) Permission/Responsibility to act “permission to do something” (p. 7) **Behavioral**: *Provision of informal support* **6 months** Taking actions “safety planning” (p. 8) Instruments: unvalidated, researcher developed
Drumm et al. (2018) Before and After USA	Informal supporters (Faith Leaders) Total sample *n* = 104 (pre and post-test), *n* = 72 (1 year follow up) Female (~4%) and Male (96%), Ethnic/racial minority (~16% Hispanic) and majority (~84% White)	Compulsory attendance **Delivery**: Face to Face **Format**: Workshop Duration: 4 hrs **Content**: Understanding and responding to DVA **Trainer**: Researchers/Unspecified	**Cognitive** *Awareness & understanding of DVA* Immediate Misunderstanding of abuse dynamics reduced from mean score of 2.42 to 1.79 (*p* ≤ .05) **12 months** Misunderstanding of abuse dynamics reduced from mean score of 2.42 to 2.08 (*p* ≤ .05) *Knowledge of support services* **Immediate** Resource Knowledge scale mean percent agreement increased from 50% to 87% (*n* = 104, mean score pre 3.31, post 4.29, *p* = <.05). **12 months** Improvements in resource knowledge (*n* = 72, mean score pre 3.31, 1 year follow-up 4.02, *p* ≤ .05). *Confidence and motivation* Immediate improvements in the ability and motivation (*n* = 104, mean score pre 4.25, post 4.66, *p* ≤ .05) **12 months** Improvements in confidence and motivation (*n* = 72, mean score pre 4.25, 1 year follow up 4.34, *p* ≥ .05). **Behavioral:** *Provision of informal support* **12 months** Increase in the number of actions taken by clergy to respond to DVA in their congregations (Mean difference: pre 1.49; 1 year 1.60, *p* = .597) Instruments: researcher developed
Flanigan (2011) Before and After, Qualitative interviews, Quantitative surveys Canada	Informal supporters (Workplace colleagues; Community members) Total sample *n* = 1,899 (surveys), *n* = 60 (interviews), Female and Male (Unspecified gender ratio), Ethnicity not reported	Voluntary attendance **Delivery**: Face to Face **Format**: Presentation/lecture **Duration**: one off, 1 hr session for workplace; unspecified for community **Content**: “basic information on risk factors and warning signs”; unspecified for community training **Trainer**: public service facilitators in workplaces (who had received 2-day training session to deliver presentation); unspecified for community training Multi-component intervention	**Cognitive** *Awareness & understanding of DVA* Immediate Awareness of DVA (before *M* = 3.35, *SD* = 1.08 and after *M* = 4.46, *SD* = 0.68, p < .001) (Workplace colleagues) Of the survey respondents, 89.2% (response rate of 36%, *n* = 5,268 trained individuals) felt prepared to identify the warning signs and risk factors of woman abuse following the training presentation (Community members) Recognizing DVA: “domestic violence happens more than I thought,” “learning about warning signs” (p. 48). *Knowledge of support services* **Immediate** Increased feelings of preparedness to provide support to victim-survivors before (*M* = 3.45, *SD* = 1.17) and after the intervention (*M* = 4.40, *SD* = 0.76, p < .001) (Workplace colleagues) 88% of respondents from community settings felt “empowered to provide other supports” (response rate of 36%, *n* = 5,268 trained individuals) (Community members) Practical responses: “learning about . . . how to take action” (p. 48), “We have lots of new knowledge, tools, resources—we are better equipped” (p. 41) Making connections: “important information and linkages to existing community resources” *Confidence and motivation* **Immediate** Confidence: “confidence and resources” as elements to take away from training (p. 46) Permission/Responsibility to act: “everyone’s business” (p. 40) Instruments: researcher developed

Glass et al. (2010) Before and After, Qualitative focus groups, Quantitative surveys USA	Informal supporters (Workplace colleagues) Total sample *n* = 53 Female (~50%) and Male (~50%), Ethnicity not reported	Compulsory attendance: Not reported **Delivery**: Online **Format**: Video **Duration**: one off, 1 hr session **Content**: The impacts of abuse, strategies used by abusers to control victim, support needs of victims, methods for supporting/helping victims **Trainer**: Automated, online (no human trainer)	**Cognitive** *Awareness & understanding of DVA* **Immediate:** Paired *t*-tests identified improvements in knowledge of DVA (p < .001, effect size = 2.29) andAnalysis of Variance (ANOVA) reported significant effects (3.56) as the mean correct scores rose from 71.8% (*SD* = 0.130) to 96.1% (*SD* = 0.132) (p < .001) *Confidence and motivation* **Immediate** 67.3% agreed that training “changed my motivation to address domestic violence in the workplace” Instruments: Unvalidated, researcher developed

Pillinger (2020) Qualitative interviews, Quantitative surveys Global	Informal supporters (Workplace colleagues) Total sample not reported Gender and ethnicity not reported	Compulsory attendance in some workplaces **Delivery**: Online **Format**: Presentation/lecture, Webinars **Duration**: Not reported **Content**: recognizing the signs of domestic violence and abuse, knowing how to respond and refer **Trainer**: Unspecified but carried out in partnership with domestic abuse organizations Multi-component	**Cognitive** *Awareness & understanding of DVA* **Unspecified timing** Recognizing DVA: “Training has also been critical in helping line managers to understand domestic violence and abuse in all of its forms, including the impact of power and control, coercive control and economic abuse” (p. 7) *Knowledge of support services* **Unspecified timing** Practical responses: “how to refer to specialist services” Instruments: not reported
Prosman et al. (2014) Before and After Netherlands	Informal supporters (Community Members) Data for Victim-Survivors Total sample *n* = 43 Female (100%) Ethnicity not reported	Voluntary attendance **Delivery**: Unspecified **Format**: Unspecified **Duration**: 10 days **Content**: “The training consisted of theoretical backgrounds of Intimate Partner Violence, depression, children witnessing abuse, parenting support, practical skills on the protocols used and how to deal in emergency situations” **Trainer**: Unspecified Multi-component intervention	*Victim-Survivor* *Mental health* **4 months** Reduced depression mean score (Sympton Checklist, SCL 90, pre 53.3, *SD* = 13.6, post 34.8, *SD* = 11.5, 95% CI 14.4–22.6, *p* ≤ .001) Experience of violence and abuse **4 months** Reduced violence and abuse mean score difference (Composite Abuse Scale, CAS scores, 3.37, *SD* = 25.7, 95% CI 29.8–45.6, *p* ≤ .001) *Social support* **4 months** Increased social support mean score (Utrecht Coping List, UCL scores pre 13.2, SD = 4.0, post 15.2, *SD* = 3.5, 95% CI −2.9 to −1.0, *p* ≤ .001) Instruments: validated tools Composite Abuse Score, Symptom Checklist, 90, Utrecht Coping List
Ross (2013) Randomized Controlled Trial, Qualitative questionnaires USA	Informal supporters (Peers) Total sample *n* = 13 Female (100%), Ethnic/racial minority: African American (20%), African (26.6%), Caribbean (40%). Ethnic/racial majority: White (6.7%)	Voluntary attendance **Delivery**: Face to face **Format**: Unspecified **Duration**: 18 hrs in total (9 × 2 hrs sessions) **Content**: Understanding DVA and support/services **Trainer**: Researcher/unspecified	**Cognitive** 4 weeks *Awareness & understanding of DVA*: Increase in the mean pre-test (18.0, *SD* = 2.5) and mean post-test (19.7, *SD* = 1.5) were significantly different, *t*(9) = −2.4, *p* = .038, *d* = 0.77. **Behavioral:** *Provision of informal support* **4 weeks** Instruments: validated Domestic Violence Self-Efficacy, Adult Self Expression Scale, Herth Hope Index
Schuler et al. (2011) Qualitative interviews and focus groups Vietnam	Informal supporters (Faith Leaders, Community members, Neighbors) Victim-Survivors Total sample *n* = 146 (interviews) Gender and ethnicity not reported	Compulsory attendance: Not reported **Delivery**: Face to Face **Format**: Workshops; unspecified **Duration**: unspecified **Content**: unspecified **Trainer**: unspecified Multi-component intervention	Informal supporter themes **Cognitive** *Awareness & understanding of DVA* **Immediate and short term (unspecified**) Recognizing DVA: “improve their knowledge about gender violence” *Knowledge of support services* **Immediate and short term (unspecified)** Practical responses: “mixed results” where “people in the supportive systems often did not know how to help women who faced GBV” (Gender Based Violence) (p. 1428) **Behavioral** **Immediate and short term (unspecified)** *Providing support*: “enabling local people to intervene” Instruments: unvalidated, researcher developed
Taft et al. (2011) Randomized Controlled Trial Australia	Informal supporters (Community members) Data for Victim-Survivors Total sample *n* = 174 (*n* = 133 1 year follow up) Female (100%), Ethnic minority and majority (Unspecified “diverse backgrounds”)	Voluntary attendance **Delivery**: Face to Face **Format**: Workshops **Duration**: Five days over five weeks **Content**: “befriending, domestic violence advocacy, working with depression, parenting support, safety and self-care” **Trainer**: Experienced supervisor (with knowledge of domestic abuse) Multi-component intervention	*Victim-Survivor mental health* **24 months after training** (12 months after intervention) Reduced depression mean scores in intervention group *(Edinburgh Postnatal Depression Scale score ≥13)* (pre 15.0, post 8.9) versus control (pre 12.9, post 9.9), adjusted difference −1.90 (95% CI −4.12 to 0.32, *p* = .09) Improved mental health mean score in intervention group (Mental Component Score-SF36, pre 28, post 38.4) versus control (pre 31.3, post 37.6), adjusted difference 2.26 (95% CI −1.48 to −6.00, *p* = .2) *Experience of violence and abuse* **24 months after training** Reduced violence and abuse mean scores in the intervention compared with the control (CAS scores, 15.9 vs. 21.8, AdjDiff −8.67, CI −16.2 to −1.15) *Physical wellbeing mean scores* **24 months after training** *(PCS-SF36: AdjDiff 2.79; CI −0.40 to 5.99)* No observed effect on Parenting stress Instruments: validated Composite Abuse Scale (CAS), Edinburgh Post-natal Depression Scale, SF-36, Parenting Stress Index Short Form (PSI-SF) Medical Outcomes Scale Short Form, (MOS-SF)
Women’s Aid (2020) Before and After, Qualitative interviews UK	Informal supporters (Community members) Total sample *n* = 645 (Sample 1, before and after evaluation forms), *n* = 424 (Sample 2 questionnaires and qualitative interviews) Female (81.5% Sample 1, 99% Sample 2), Male (18.5% Sample 1, 1% Sample 2), Non-binary (0.3% Sample 1) Ethnic minority (Sample 1 Asian/Asian British 6.5%, Black/African/Caribbean/Black British African 3.2%, Mixed ethnic 2.2%, Sample 2 16% BAME or Other White) and Ethnic majority (Sample 1 73.7% White British; Sample 2 84% White British)	Voluntary attendance **Delivery**: Face to face **Format**: Presentations/lectures; Workshops **Duration**: 12 hrs **Content**: Awareness of DVA, challenging myths, listening to victim-survivors, supporting and signposting **Trainer**: specialists in domestic abuse alongside partnership with domestic abuse organizations Multi-component intervention	**Cognitive**: *Awareness & understanding of DVA* **Immediate:** Changes in understanding of patterns of coercive control and domestic abuse (*n* = 654, with change in average rating of +1.2), the causes of DVA (e.g., before training, 41.6% of respondents believed anger, drugs, and drink are largely responsible for DVA, reducing to 12.1% after) and the gendered nature of abuse (agreement with statement that women form the majority of the victims increased from 70.9% before to 88.5% after training). Increase in agreement that “DVA is part of some cultures” (rising from 24.2% to 26.2%) *Knowledge of support services* **Immediate** Making connections: “I always give them all the information about Country Haven Women’s Aid and Women’s Aid” (p. 14) *Confidence and motivation* **Immediate** +1.5 change in average rating of (unvalidated) measure of “confidence in skills to share information and signpost a survivor to get support.” Statistical significance not tested. (Community members) **Behavioral** **Immediate and short term (unspecified)** *Provision of informal support* “Ambassadors reported having conversations addressing DVA since the Ask Me training,” “I have been sharing posts on Facebook, from Women’s Aid. . .” Instruments: unvalidated, researcher developed

DVA = domestic violence and abuse.

### Quality of Included Studies

Study designs were diverse and of variable methodological quality but judged to be “good enough” to contribute toward synthesis. Eight included studies used experimental (*n* = 3) or quasi-experimental designs (*n* = 5). One RCT fulfilled all MMAT criteria (Choi et al., 2019), and two RCTs met 40% of the criteria, demonstrating strengths in randomization and adherence of participants to their assigned condition (Ross, 2013; Taft et al., 2011). Three of the five quasi-experimental designs were judged to be methodologically robust, meeting between 60% (Prosman et al., 2014) and 80% of the MMAT criteria (Drumm et al., 2018; Glass et al., 2010). The two remaining before and after studies were from gray literature sources and did not meet any of the MMAT criteria, suffering from a range of weaknesses including incomplete outcome data and confounding factors (Flanigan, 2011; Women’s Aid, 2020). Both studies, however, were judged to offer authority, relevance and significance based on the AACODS tool. The three remaining studies included two qualitative studies, fulfilling 100% (Schuler et al., 2011) and 40% (Debbonaire et al., 2011) of the MMAT criteria for qualitative studies. The final included study met 0% the MMAT mixed methods criteria (Pillinger, 2020) but, as gray literature, was judged as providing significant and authoritative contributions to the knowledge base.

### Educational/Training Interventions

The interventions were tailored toward different settings and types of informal supporter. Workplace training (*n* = 4) targeted supervisors or managers with one-off sessions, typically lasting 60 min, delivered by trained professionals (Debbonaire et al., 2011; Flanigan, 2011), automated programs (Glass et al., 2010) or unspecified trainers (Pillinger, 2020). Most workplaces (*n* = 3) had partnered with a domestic abuse organization in developing and/or delivering the training. Involvement in the training was voluntary in two workplaces (Debbonaire et al., 2011; Flanigan, 2011), compulsory for managers in particular settings (Pillinger, 2020) or unspecified (Glass et al., 2010). Presentations/lectures or videos were the main method of delivering content and one intervention also used webinars (Pillinger, 2020). Training for informal supporters who had volunteered from the wider community (*n* = 5) tended to have a longer duration than workplace training, consisting of two (Women’s Aid, 2020), five (Taft et al., 2011) or 12 days (Prosman et al., 2014). Where specified, the method of delivery was more interactive than delivery in the workplace. Compulsory training for Faith Leaders (*n* = 2) was delivered in sessions ranging from 1.5 to 4 hrs in total. The methods of delivery included online presentations/lectures (Choi et al., 2019) and face-to-face workshops (Drumm et al., 2018). Training for peers (*n* = 1) consisted of nine, 1-hr sessions that focused on DVA and sources of support/response. These sessions were delivered in person.

Across all interventions, the curricula were broadly similar focusing on “basic information on risk factors and warning signs” of abuse (Flanigan, 2011), examining the impacts on victim-survivors, businesses, and wider community, and considering the support needs of victim-survivors. Guidance was also provided on how to respond and/or how to refer to specialist services. Of the education/training interventions that were studied, seven were implemented alongside changes in the wider setting including, for example, a new workplace domestic abuse policy (Debbonaire et al., 2011), or the development of a community-based support system for victim-survivors (Women’s Aid, 2020).

### Outcomes

Frequently measured outcomes included informal supporters’ knowledge and awareness of DVA, knowledge of support services, and/or confidence and motivation in responding (*n* = 9). Five of the studies also reported quantitative or qualitative data pertaining to subsequent actions taken by informal supporters. There were no studies that reported data on informal supporters’ emotional and practical ability to respond (such as knowledge of self-care strategies). Data were mainly self-reported by the informal supporters, with only one study using validated tools to assess outcomes for informal supporters. Most data were collected in the short term (immediately, 3 or 6 months after training). Two studies reported outcomes for victim-survivors, using validated, standardized tests.

### Informal Supporters

#### Awareness and Understanding

All experimental or quasi experimental studies (*n* = 6) reported improvements in informal supporters’ awareness and understanding of DVA immediately, 3 months and/or 12 months after education/training (see Table 1). There were immediate, statistically significant, increases in awareness (Flanigan, 2011) and knowledge (Glass et al., 2010) of DVA for workplace managers who had attended training. Studies also reported immediate improvements in Faith Leaders’, peers’ and community members’ understanding and knowledge of DVA (Drumm et al., 2018; Ross, 2013, Women’s Aid, 2020). Of the respondents to a community survey, 89.2% (response rate of 36%, *n* = 5,268 trained individuals) felt prepared to identify the warning signs and risk factors of woman abuse following the training presentation (Flanigan, 2011). Three months after training, statistically significant improvements in attitudes toward DVA were reported for trained Faith Leaders as compared to those who were untrained (Choi et al., 2019). Knowledge of DVA also improved in this group but this was not statistically significant. In the longer term, 12 months after training, statistically significant improvements in understanding of DVA were reported compared to baseline. This improvement was smaller than immediate impacts so the authors recognize that there “was a measurable amount of forgetting of what was learned in the training” (Drumm et al., 2018, p. 86) but evidence remains that training can contribute to longer term improvements in understanding of DVA (Drumm et al., 2018).

Five studies reported qualitative data on participants’ views about the impacts of training on their awareness and understanding of DVA (Debbonaire et al., 2011; Flanigan, 2011; Pillinger, 2020; Schuler et al., 2011; Women’s Aid, 2020). These studies highlighted that training (alongside the introduction of policy in some cases) served to make the issue of DVA more visible, and improved understanding of the prevalence of DVA: “domestic violence happens more than I thought” (Flanigan, 2011, p. 48). Participants reported that training supported “the development of skills in recognizing the signs of domestic violence and abuse” and so improved understanding of DVA “in all of its forms, including the impact of power and control, coercive control and economic abuse” (Pillinger, 2020, p. 7).

#### Knowledge of Support/How to Respond

Following training, Faith leaders and workplace managers improved their knowledge of how to respond to victim-survivors of DVA (*n* = 3). Studies reported statistically significant improvements in clergies’ knowledge of resources immediately (Drumm et al., 2018), 3 months (Choi et al., 2019) and 12 months after training (Drumm et al., 2018). In the workplace, there were immediate improvements in employees’ feelings of preparedness to provide support “e.g., empathetic listening, not blaming” to victim-survivors after the training sessions (Flanigan, 2011). Post intervention evaluations also identified that 88% of respondents from community settings felt “empowered to provide other supports” after training (response rate of 36%, *n* = 5,268 trained individuals) (Flanigan, 2011, p. 10).

Qualitative data from five studies highlight the importance of training for equipping informal supporters with knowledge about how to practically respond: “we are better equipped” (Flanigan, 2011, p. 41) with knowledge of “how to refer to specialist services” (Pillinger, 2020) and “how to help” (Debbonaire et al., 2011, p. 10). Participants also highlighted that training fostered connections to wider resources including “other policies and work tools” (Debbonaire et al., 2011), “linkages to existing community resources” (Flanigan, 2011) and local support organizations (Women’s Aid, 2020, p. 14).

#### Confidence and Motivation

Following training, members of the clergy reported statistically significant, immediate improvements in their ability and motivation to act (Drumm et al., 2018). Longer term improvements at 3 and 12 months were also reported but these were not statistically significant (Choi et al., 2019; Drumm et al., 2018). The confidence of Faith Leaders to respond to victim-survivors significantly declined 1 year after training (Drumm et al., 2018). For community members, one study found that training was associated with increased “confidence in skills to share information and signpost a survivor to get support” although statistical significance was not tested (Women’s Aid, 2020). Within the workplace, one study reported that on completion of training for supervisors in the workplace, 67.3% (*n* = 53) agreed that training “changed my motivation to address domestic violence in the workplace” (Glass et al., 2010, p. 171).

Qualitative studies reported that training played an important role in developing the confidence of employees to support victim-survivors in the workplace (Debbonaire et al., 2011; Flanigan, 2011). Managers recognized that the training gave them permission/responsibility to act (Debbonaire et al., 2011, p. 7) as DVA was “everyone’s business” (Flanigan, 2011, p. 40).

#### Behavioral

Two quantitative studies assessed the impacts of training for Faith Leaders’ subsequent behavior. Three months after training, there was an increase in the number of actions taken by clergy who had received training, compared to those who had not, but the differences between these groups did not reach statistical significance (Choi et al., 2019, p. 30). Similarly, at 12 months after training, Faith leaders took more actions to respond to DVA in their congregations than before their training, but these differences were not statistically significant (Drumm et al., 2018).

Four studies report qualitative accounts of actions taken by informal supporters following the training (Debbonaire et al., 2011; Pillinger, 2020; Schuler et al., 2011; Women’s Aid, 2020). In the workplace, one respondent identified that the training information helped “to make a difference” and lead to improvements in her efforts to respond to colleagues (Debbonaire et al., 2011, p. 8). Respondents detailed a range of actions that they’d taken to support colleagues such as “safety planning” and “giving time off to see solicitors.” In the community setting, Schuler et al. (2011) associated the intervention (which included training and additional components) with “enabling local people to intervene . . . (more quickly, systematically, and effectively than was traditionally the case).” Following training of community members, participants reported having conversations and sharing information with survivors and wider networks (Women’s Aid, 2020).

### Victim-Survivors

Two experimental or quasi-experimental studies assessed the impacts of a mentor mother intervention, part of which included training for the volunteer mentor (Prosman et al., 2014; Taft et al., 2011). Both studies reported statistically significant reductions in violence and abuse experienced by victim-survivors who had been mentored. There was weaker evidence for other outcomes but both studies found reductions in depression and improvements in their social support/networks for victim-survivors who had been mentored.

### Theoretical Framework

Overall, the included studies ratified the theoretical understanding of how education/training interventions may prompt informal supporters to respond to victim-survivors. Cognitive and behavior outcomes for informal supporters were appropriate, except for “emotional ability to respond” which was not assessed by studies or reported as a part of the training curricula. Qualitative data from included studies demonstrated the interaction of cognitive outcomes (e.g., perceptions of knowledge of DVA interacts with confidence/motivation to respond) and so highlight the value of conceptualizing outcomes in this way. However, studies did not provide sufficient data to confirm or challenge the expected pathways for informal supporters’ behavioral outcomes or subsequent victim-survivor outcomes. Findings did not, for example, shed light on what types of support informal supporters would provide and/or whether this would be considered helpful and/or sustained over the longer term.

### COM-B Mapping

Table 2 maps the outcome categories identified in this review on to the COM-B framework and illustrates the extent of included quantitative evidence for each construct, based on the timing of the outcome measures and whether they tested/reached statistical significance. Of the six included experimental or quasi-experimental studies, half showed statistically significant improvements in all three COM-B constructs, capability, opportunity, and motivation (Choi et al., 2019; Drumm et al., 2018; Women’s Aid, 2020). This means that there is evidence that education/training fulfills the essential conditions for changing the behavior of informal supporters.

**Table 2.** Extent of Quantitative Evidence for Capability, Opportunity, and Motivation (COM), by Timing of Outcome.

Timepoint	Total Number of Studies Reporting Improvements	Studies	Number of Studies Reporting Statistically Significant Improvements
Capability (All outcomes)			
Immediate	5	Drumm et al. (2018), Flanigan (2011), Glass et al. (2010), Ross (2013), Women’s Aid (2020)	4
Three months	1	Choi et al. (2019)	1
Twelve months	1	Drumm et al. (2018)	1
Opportunity (Awareness and understanding of DVA)			
Immediate	5	Drumm et al. (2018), Flanigan (2011), Glass et al. (2010), Ross (2013), Women’s Aid (2020)	4
Three months	1	Choi et al. (2019)	1
Twelve months	1	Drumm et al. (2018)	1
Motivation (Confidence and motivation to respond)			
Immediate	2	Drumm et al. (2018), Women’s Aid (2020)	1
Three months	1	Choi et al. (2019)	0
Twelve months	1	Drumm et al. (2018)	0

DVA = domestic violence and abuse.

## Discussion

This mixed method review presents a theoretical model for understanding the impacts associated with training tailored toward informal supporters (Figure 1) together with underpinning data derived from current research. The evidence base is limited but sufficient to recognize that educational activities lead to improvements in knowledge and attitudes in the short term (See Table 3). Such findings are consistent with wider systematic reviews that have found that educational interventions have improved the knowledge and resources of informal supporters in the workplace (Adhia et al., 2019) and community settings (Ogbe et al., 2020). These findings also echo reviews of DVA training in professional settings (Serrano-Montilla et al., 2021; Turner et al., 2017; Zaher et al., 2014) where such programs are deemed essential for enabling healthcare professionals to respond to victim-survivors (Ambikile et al., 2022). This review highlights the potential of educational activities for encouraging positive, rather than negative, social reactions to disclosures of abuse (Dworkin et al., 2019; Ullman, 2021), namely to listen empathetically and offer practical help (Sylaska & Edwards, 2014).

**Table 3.** Critical Findings.

• There is statistically significant evidence that training/educational activities aimed at informal supporters improves their *knowledge and attitudes* in the short term. • Trained informal supporters recognize the importance of education for building understanding and equipping them with skills to respond to victim-survivors of DVA. • Training/educational activities prompt informal supporters to respond to victim-survivors, as well as enhancing their capacity and motivation to do so. This increases the likelihood that informal supporters will *take action* to support victim-survivors of abuse. We don’t know, however, what type of support they will provide and/or whether it would be judged to be helpful by victim-survivors.

DVA = domestic violence and abuse.

However, there are limited data on longer term effects on knowledge and attitudes (echoing the findings of other reviews such as Ogbe et al., 2020; Turner et al., 2017). Improvements were reported by one study 12 months after a one-off training session, but these were not statistically significant and there were observable lapses in supporters’ knowledge over time (Drumm et al., 2018). This points to the need for multiple training sessions, over an extended period, for providing sustained improvements, especially in attitudes (Sammut et al., 2021; Sprague et al., 2018; Turner et al., 2017). The features of the included educational interventions, such as the involvement of DVA organizations in the training and the provision of practical resources, have been associated with effective DVA training in professional settings (Sprague et al., 2018) and so may also be important in this context.

While this review advances our understanding of the impacts of training for informal supporters, the small number of studies and limitations in reporting mean that this article can only provide a partial picture. The review did not find any data on the impacts of training on informal supporters’ emotional/practical ability to respond. Yet, it is vitally important to consider such outcomes. Friends, family, colleagues, and neighbors report emotional stress/fatigue when supporting someone experiencing DVA (Gregory, Williamson et al., 2017) and providing emotional care is considered an important pillar of informal support (Sylaska & Edwards, 2014). Education about emotional well-being/self-care, for example, should therefore be an essential part of training for informal supporters. However, emotional wellbeing was rarely considered in the curricula of the included interventions (only one intervention covered the topic of “self-care” Women’s Aid, 2020). This lack of consideration may reflect the type of supporters and settings targeted by the training. Many studies (*n* = 7) evaluated training for individuals who offer informal support in professional, public settings such as workplaces or churches. Training curricula emphasized resources for referral, practical assistance, and institutional responsibilities (such as the “laws regarding employer responsibilities,” see Glass et al., 2010) rather than emotional forms of support. Indeed, the type of support provided in professional/public settings may differ from the assistance provided by supporters in private/intimate settings (such as friends or family) (Goodman, Banyard, et al., 2016). Therefore, the content of the training may need to be tailored to the type and quality of relationship between informal supporter and victim-survivor (Gregory, Feder et al., 2017), and the settings in which support is provided. This would bring further nuance to the theoretical framework outlined above (Figure 1).

While there was limited quantitative data on confidence and motivation, qualitative accounts suggest that training plays an important role in boosting confidence, as does wider evidence (Sammut et al., 2021). Minimal data on the demographic composition of study samples mean that it is difficult to ascertain the impacts across gender, ethnicity, and age even though we know that these play an important role in mediating the provision and uptake of informal social support (Bundock et al., 2020; Sultana et al., 2022).

The review identified limited data to directly evidence the link between shifts in informal supporters’ knowledge and attitudes, and their subsequent actions/provision of support. This reflects wider trends where evaluations of DVA training in professional contexts have rarely reported subsequent behavioral outcomes for users or patients (Sprague et al., 2018). Using the COM-B framework, this review identified evidence that education/training for informal supporters meets the three essential conditions for behavior change. This means that, following education/training, we can reasonably expect that informal supporters will change their behavior toward victim-survivors of DVA (See Table 3). Indeed, qualitative data from four included studies suggests that individuals recognized the importance of training for prompting and shaping their subsequent actions. However, we do not know if such changes in behavior/the provision of informal support will translate into improved outcomes for victim-survivors (Sammut et al., 2021). As illustrated in Figure 1, the actions of informal supporters could potentially lead to less helpful responses, with adverse outcomes. However, this review identified data, albeit limited, to suggest that informal support interventions with a significant training component will lead to improved outcomes for victim-survivors. These findings are based on mentoring interventions, where training was only one component, so it is difficult to assess the role of educational activities in delivering improved outcomes. Yet, these findings are also consistent with wider evidence that associates positive forms of informal support with improved outcomes (Sylaska & Edwards, 2014).

### Limitations of the Evidence Base

The systematic review highlights that the evidence base is small and relatively under-developed. While there are diverse educational activities and/or training interventions targeting informal supporters, there are few studies designed to monitor or evaluate the impacts of these. The reports identified by this review therefore present a partial picture, with current research focusing on the education of specific types of informal supporters (employees, Faith Leaders, peers, and community members) and not others (friends or family members). The studies included mainly female samples with limited reporting on the age or ethnicity of the informal supporters. Research suggests that the use and provision of social support varies by demographic characteristics such as gender, ethnicity, and age (Bundock et al., 2020; Ragavan et al., 2020; Sylaska & Edwards, 2014), and so future research should aim to analyze diverse samples. The evidence base was predominantly drawn from North American or Global North contexts. This is a common trait of systematic reviews in the field of DVA interventions (Trabold et al., 2020) but inhibits a global perspective. This may be partly due to the selection of English language reports. Further, the evidence base is relatively old with only four included studies published within the last 5 years. This is consistent with findings of other reviews of informal support interventions and points to the dearth of research in this area (Konya et al., 2020; Ogbe et al., 2020).

The review found limited data on behavioral impacts of training/education as few studies aimed to collect such measurements. Moreover, it is difficult to appreciate the longer-term impacts of education/training for informal supporters as few studies measure outcomes at longer time points. More broadly, the studies provided scant detail on the educational intervention (such as the curriculum content and pedagogical approach) and/or specifics about the sample/population (such as ethnicity). There is also a lack of evidence on the subsequent impacts on victim-survivors. Therefore, there are several recommendations for improving research in this field (see Table 4).

**Table 4.** Recommendations for Practice, Policy, and Research.

Practice	• Continue to develop and support training/educational interventions that target informal supporters in workplaces, community settings and beyond. • Build on existing practice, alongside research evidence, to develop training to maximize the likelihood of positive outcomes. • Evaluate interventions and share learning across the sector. • Develop interventions aimed at a wide range of informal supporters (including friends and family).
Policy	• Recognize the key role played by informal supporters in responding to DVA, as part of holistic, societal-wide response to DVA. • Harness the potential of informal social networks by investing in education/training to support such groups, in collaboration with wider DVA sector. Provide funding to organizations that develop, and evaluate, training for informal supporters.
Research	• Monitor and evaluate existing educational/training activities aimed at informal supporters. Alongside academic research, this requires support for non-governmental organizations/voluntary sector to develop and apply methods for understanding and assessing the impact of their activities in this area. • Use study designs that evaluate the impacts of the training/education alongside designs that build understanding of the intervention. • Ensure full and transparent reporting of the intervention and research processes. • Evaluate the impacts of training for diverse population groups (all genders, diverse ethnic and age groups) in wider geographical contexts (beyond the Global North). • Measure behavioral outcomes for informal supporters and explore the nature of the support provided, and whether perceived to be helpful by victim-survivors. • Measure the extent to which training develops informal supporters’ practical and emotional capacity to respond. • Measure outcomes for victim-survivors.

DVA = domestic violence and abuse.

### Policy and Practice Recommendations

Within the UK and internationally in the Global North, national policy documents acknowledge the importance of informal social support for victim-survivors of DVA (e.g., Home Office, 2016; Australian Institute of Health and Welfare; Government of Canada Department of Justice). Policy initiatives targeting informal social support, however, are limited as government-sponsored interventions primarily focus on the delivery of support through formal channels (such as criminal justice or health alongside non-governmental organizations). This review re-affirms the importance and potential of informal social networks for supporting victim-survivors of DVA as part of a whole-system response (Goodman, Banyard, et al., 2016). Informal networks provide unique opportunities to respond to victim-survivors, including groups who are unwilling/unable to disclose to formal services (Femi-Ajao et al., 2020; Sultana et al., 2022). Training programs can help to empower these individuals, who likely perform a supporting role already (especially female friends, family, and colleagues, McKenzie et al., 2020) and so policy interventions should seek to invest in educational activities to meet the needs of these groups. This review highlights the importance of working alongside DVA experts in the development and delivery of such training. Sectors that already deliver education/training should aim to further develop their interventions, drawing on research evidence, to maximize the likelihood of positive outcomes (see Table 4).
